# Persistently high HIV incidence among men who have sex with men and people who inject drugs attending integrated care centres in India: a longitudinal assessment of clinic‐based data

**DOI:** 10.1002/jia2.26361

**Published:** 2024-09-20

**Authors:** Allison M. McFall, Mihili P. Gunaratne, Lakshmi Ganapathi, A. K. Srikrishnan, C. K. Vasudevan, Santhanam Anand, David D. Celentano, Sunil S. Solomon, Shruti H. Mehta, Gregory M. Lucas

**Affiliations:** ^1^ Department of Epidemiology Johns Hopkins Bloomberg School of Public Health Baltimore Maryland USA; ^2^ Division of Pediatric Infectious Diseases Massachusetts General Hospital Boston Massachusetts USA; ^3^ YR Gaitonde Centre for AIDS Research and Education Chennai India; ^4^ Department of Medicine Johns Hopkins University School of Medicine Baltimore Maryland USA

**Keywords:** delivery of healthcare, HIV testing, incidence, India, integrated, intravenous, sexual and gender minorities, substance use

## Abstract

**Introduction:**

Globally, there have been significant declines in HIV incidence over the past two decades, but this decline is slowing, and in some settings, declines have stalled or are growing—particularly where epidemics are concentrated in key populations (KPs). Understanding temporal changes in HIV incidence among KP is critical yet, due to logistical constraints, there are few sources of longitudinal incidence data, particularly among KP.

**Methods:**

We present HIV incidence rates from June 2014 to December 2022 among cisgender men who have sex with men (MSM) and people who inject drugs (PWID) attending community‐based integrated care centres (ICCs) in 15 Indian cities. ICCs, established between 2014 and 2017, provide HIV testing and other services to MSM (eight sites) or PWID (eight sites). Client HIV testing data were included in the analysis if they had ≥2 tests and were not positive on the first test. We calculated incidence rates per 100 person‐years (PY), stratified by KP, city/site and year. Poisson regression explored associations of incidence with time, age, gender (PWID only) and ICC use.

**Results:**

From June 2014 to December 2022, 13,501 clients (5722 MSM, 7779 PWID) had ≥2 HIV tests over a median of 1.8 years. There were a total of 1093 incident HIV acquisitions. Overall incidence rates for MSM and PWID were 1.9/100 PY (95% CI: 1.7−2.2) and 4.1 (3.9−4.4), respectively. Among MSM sites, incidence ranged from 0.4 to 3.5 and in PWID sites from 0.6 to 17.9. From adjusted models, incidence increased by 17% annually among MSM. Among PWID, incidence increased by 11% annually up until 2020 and then decreased by 29% after 2020; when excluding the outlier of New Delhi, incidence was stable among PWID. MSM and PWID 21−25 years old had the highest risk of HIV and among PWID, those more consistently engaged in medication for opioid use disorder were at the lowest risk.

**Conclusions:**

While there was substantial geographic variability, MSM and PWID engaged in a free community‐based clinic experienced persistently high HIV incidence (>2/100 PY). KP in low‐ and middle‐income countries should be a focus when considering novel strategies such as long‐acting pre‐exposure prophylaxis to curtail incidence.

## 1 INTRODUCTION

Globally, there have been dramatic declines in HIV acquisitions—reduced by over 50%—since the peak in 1996 [1] but recently, these improvements have slowed, especially since the COVID‐19 pandemic. HIV treatment coverage is at its slowest growth over the past decade and there were more than 1 million HIV acquisitions than global targets in 2021 [2]. Additionally, in regions outside of sub‐Saharan Africa, where epidemics are concentrated in key populations (KPs), HIV incidence declines have stalled [3]. Some regions—Eastern Europe, central Asia, Middle East and Latin America—have experienced increasing annual HIV acquisitions from 2015 to 2021 [2]. KP—including people who inject drugs (PWID) and men who have sex with men (MSM)—bear a disproportionate burden of HIV with risks 35 and 28 times higher, respectively, than the general adult population [1]. In India—a country with the third largest number of people living with HIV globally [4]—general population prevalence is relatively low (0.21%) [5] but KPs such as MSM and PWID experience a high HIV burden—2.7% among MSM and 6.3% among PWID in 2017 [6]. Thus, these global estimates and trends conceal considerable heterogeneity by region, country and population.

Understanding temporal changes in HIV incidence among KP is critical to addressing the epidemic and responding effectively. The UNAIDS Fast‐Track 2030 targets include substantial reductions in HIV acquisitions and emphasize a geographically targeted and human‐rights approach, prioritizing intensified coverage of combination prevention interventions among KP [7]. Yet, due to limited funding and complex logistics, community‐based longitudinal incidence data, particularly among KP, are relatively scarce. We present HIV incidence rates from June 2014 through December 2022 among MSM and PWID attending community‐based population‐specific care centres in 15 Indian cities.

## 2 METHODS

### 2.1 Integrated care centres

Integrated care centres (ICCs) were established in 15 Indian cities for two clinical trials [8−10]; eight ICCs were established starting in mid‐2014 (MSM: Bangalore, Belgaum, Hyderabad, Visakhapatnam; PWID: Aizawl, Bilaspur, Dimapur, Ludhiana) and the remaining in mid‐2017 (MSM: Bhopal, Madurai, New Delhi‐MSM, Vijayawada; PWID: Amritsar, Churchandpur, New Delhi‐PWID, Kanpur). ICCs provide general health and HIV‐related services to either PWID or cisgender MSM (transgender [TG] women were not targeted as they typically receive services from TG‐focused centres/organizations).

All ICC services were provided free‐of‐charge and included onsite HIV counselling and testing, general health screenings (e.g. tuberculosis), condoms, sexually transmitted infection screening and treatment, counselling (e.g. risk reduction) and navigation to HIV care/treatment at government centres for those living with HIV. Additional PWID‐specific services included daily observed medication for opioid disorder (MOUD), field‐based needle/syringe distribution and hepatitis C antibody testing. ICCs utilized an electronic database to register clients and track service utilization and lab results for clients over time. Biometrics identified unique ICC clients. Additional details on ICC services have previously been published [8, 9, 11].

### 2.2 HIV testing at ICCs

Most ICC clients underwent HIV testing at ICC registration unless they had documentation they were living with HIV. HIV testing protocols varied across sites but ICCs either conducted the rapid point‐of‐care 3‐test protocol (specified by Indian guidelines) or conducted a single point‐of‐care screening test at the ICC, followed by confirmatory testing at a government facility, if positive. For those negative, outreach staff contacted and encouraged clients to return for repeat testing every 6 months according to Indian guidelines.

### 2.3 Statistical analysis

We used ICC data starting from the establishment of the ICCs (either 2014 or 2017, depending on the site, as indicated above) through 31 December 2022. We abstracted client HIV testing data—date and test results—and client age, gender and number of ICC/MOUD visits. Client testing data were included in analyses if they had ≥2 HIV tests and were not positive on the first test. The Johns Hopkins Medicine Institutional Review Board granted a waiver of consent for these analyses.

Self‐reported risk behaviour data were collected during HIV counselling and testing services and reflected behaviours in the prior 6 months. Person‐time was accrued between HIV test dates with an exception for those with a positive result, for whom the seroconversion date was estimated as the midpoint between the last negative and first positive test and person‐time was calculated accordingly. Incidence rates per 100 person‐years (PY) were stratified by KP, site and year. To visualize temporal trends in incidence, we used line graphs per population group and site, with a 2‐year moving average to smooth short‐term fluctuations. Additionally, separately for MSM and PWID, multi‐level Poisson regression models with random intercepts for site were used to assess the association of incidence with time as calendar year, age, gender (PWID only), any ICC use (average number of ICC monthly visits for any service [including MOUD for PWID]) and MOUD use (average number of monthly visits for MOUD, PWID only). Correlates were included in univariable and multivariable models resulting in unadjusted and adjusted incidence rate ratios (aIRRs). Modelling of time included an exploration of splines and determination of the best fit using the Akaike information criterion and Bayesian information criterion. As a sensitivity analysis for the PWID model, New Delhi (an outlier in incidence rate) was excluded to assess whether this one site predominantly drove associations. Data analyses were completed using Stata (StataCorp. 2021. Stata Statistical Software: Release 17. College Station, TX: StataCorp LLC.) and statistical significance was considered as *p*<0.05.

## 3 RESULTS

**Figure 1:**
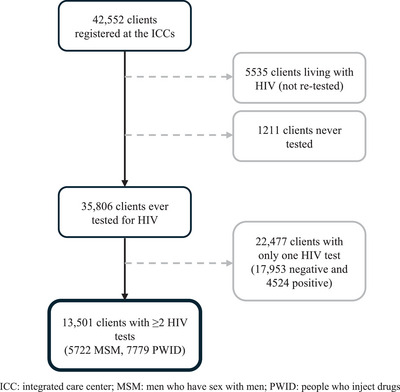
Flow diagram of HIV incidence analytical sample of ICC clients across 15 Indian cities.

Between June 2014 and December 2022, a total of 42,552 clients registered at ICCs of which 1211 were never tested and 5535 were living with HIV at the time of registration and thus not re‐tested (Figure 1). Among 35,806 clients ever tested for HIV at the ICC, 22,477 had only one test of whom 17,953 were negative and 4524 were positive. For the analytical sample, a total of 13,501 clients (5722 MSM, 7779 PWID) had ≥2 HIV tests with a median follow‐up time of 1.8 years (interquartile range [IQR] 0.9−3.6). Median (IQR) number of total HIV tests was 3 (2−5) and median time between tests was 7 months (5−8 months). Median age at the first test was 27 years and 7% of PWID were women and 0.3% TG. Among MSM, 66% had condomless anal sex with a man in the prior 6 months at their first HIV test. Among PWID, 64% had injected drugs in the prior 6 months and 40% had shared injecting paraphernalia (i.e. needles and/or syringes) in the prior 6 months at their first test. Among MSM negative on their first test, those who did not return for testing were older (≥25 years 56% vs. 45%) and less likely to report condomless sex (66% vs. 71%). For PWID clients negative on their first test, those who did not return for testing were older (≥25 years 65% vs. 56%) and more likely to be female (10% vs. 7%), unemployed (38% vs. 31%) and report sharing injecting paraphernalia more frequently (44% vs. 39%), compared to those who returned.

There were a total of 1093 incident HIV acquisitions (227 MSM, 866 PWID) among 13,501 clients. The overall incidence rate for the full‐time period was 1.9 per 100 PY (95% confidence interval [CI]: 1.7−2.2) for MSM and 4.1 (95% CI: 3.9−4.4) for PWID. Among MSM sites, incidence ranged from 0.4 (Belgaum) to 3.5 (Bangalore) per 100 PY and in PWID sites, from 0.6 (Dimapur) to 17.9 (Delhi).

**Figure 2:**
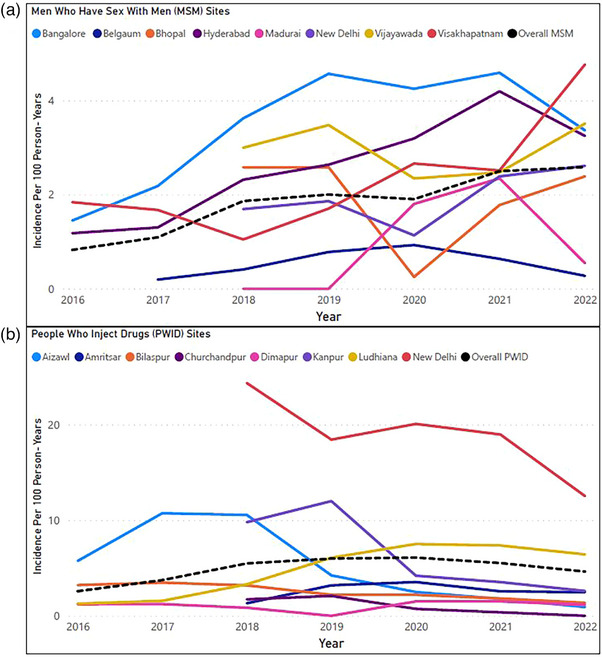
HIV incidence rates per 100 person‐years among men who have sex with men and people who inject drugs attending integrated care centres in India 2015−2022, by city and year using a 2‐year moving average. *Note*: MSM in panel A and PWID in panel B. The two panels have different Y‐axis scales due to differing ranges of incidence rates between MSM and PWID sites. Half of the sites were established later—in 2017; these sites have lines beginning in 2018 (i.e. an average of 2017 and 2018).

Figure 2 shows HIV incidence per site and population group over time, smoothed using a 2‐year moving average. For 2014, there was only ∼6 months of follow‐up time and no acquisitions, therefore, analyses by year excluded 2014. For MSM overall, there was a gradual increase in incidence over time (0.8/100 PY in 2016 to 2.6 in 2022) though there was variability in the trajectory for individual sites. For PWID overall, there was an increase initially (2.6 in 2016 to 5.5 in 2018), followed by a more stable period, then a slight decline (6.1 in 2020 to 4.6 in 2022). Individual PWID sites tended to follow this similar trajectory though there is significant variability in the magnitude (i.e. New Delhi).

Table 1 presents temporal trends and the association of age, gender and ICC/MOUD use with incidence. In multivariable analysis among MSM, incidence increased by 17% annually. Among PWID, incidence increased by 11% up until 2020 and then decreased by 29% annually after 2020. Among both PWID and MSM, those 21−25 years old had the highest incidence (aIRR compared to 18−20 years: 1.32 for PWID and 1.56 for MSM) and then incidence generally declined with age. Among PWID, those identifying as TG had 13.4 times higher incidence compared to men, while women had lower incidence (aIRR: 0.53) and more frequent MOUD use was associated with lower incidence (aIRR of >10 average MOUD visits/month vs. none: 0.40); any ICC use and MOUD use were highly collinear as the primary service used at PWID ICCs is MOUD. ICC use was not associated with incidence among the MSM. In the PWID sensitivity analysis excluding New Delhi, associations with other covariates were unchanged; however, the best fit for year was modelling without a spline, resulting in a 6% annual increase in incidence in univariable (aIRR: 1.06, 95% CI: 1.02–1.10) and no significant association with year in multivariable models (aIRR: 1.00, 95% CI: 0.96–1.05).

**Table 1:** Temporal trends and correlates of HIV incidence among men who have sex with men and people who inject drugs attending integrated care centres (ICCs) in India

	Incidence rate ratio (95% CI)	Adjusted incidence rate ratio (95% CI)
Men who have sex with men		
Time—by 1 year increase	1.17 (1.09, 1.27)	1.17 (1.08–1.26)
Age (years)		
18−20	Reference	Reference
21−25	1.63 (1.14–2.32)	1.56 (1.10–2.22)
26−30	0.88 (0.57–1.38)	0.84 (0.54–1.32)
31−35	0.84 (0.49–1.44)	0.84 (0.49–1.44)
36−40	1.05 (0.57–1.96)	1.03 (0.55–1.91)
41−45	0.92 (0.41–2.05)	0.91 (0.41–2.04)
46 and above	0.25 (0.06–1.03)	0.25 (0.06–1.02)
Average monthly ICC visits		
≤1	Reference	
>1	0.91 (0.22–3.66)	–

People who inject drugs		
Time—by 1 year increase with spline		
2015 until 2020	1.15 (1.09–1.22)	1.11 (1.05–1.17)
2020 until 2022	0.75 (0.66–0.85)	0.71 (0.63–0.81)
Gender		
Man	Reference	Reference
Woman	0.65 (0.42–1.00)	0.53 (0.34–0.82)
Transgender/hijra	11.6 (1.62–83.3)	13.4 (1.85–96.7)
Age (years)		
18−20	Reference	Reference
21−25	1.33 (1.09–1.61)	1.32 (1.09–1.61)
26−30	1.10 (0.89–1.36)	1.11 (0.90–1.37)
31−35	0.84 (0.65–1.08)	0.84 (0.65–1.09)
36−40	0.59 (0.42–0.83)	0.62 (0.44–0.87)
41−45	0.44 (0.27–0.71)	0.46 (0.28–0.76)
46−50	0.14 (0.04–0.43)	0.15 (0.05–0.48)
51−55	0.06 (0.01–0.44)	0.07 (0.01–0.48)
56 and above	0.05 (0.01–0.38)	0.05 (0.01–0.37)
Average monthly ICC visits		
≤10	Reference	–
>10	0.61 (0.51–0.72)	
Average monthly MOUD visits		
None/not on MOUD	Reference	Reference
>0 to 10	0.52 (0.45–0.61)	0.49 (0.42–0.57)
>10	0.40 (0.33–0.48)	0.40 (0.33–0.49)

Abbreviations: CI, confidence interval; ICC, integrated care centre; MOUD, medication for opioid use disorder.

## 4 DISCUSSION

While there was substantial geographic variability, throughout most of 2014−2022, both MSM and PWID engaged in a community‐based clinic where no‐cost HIV testing, counselling and other preventive services were provided experienced high HIV incidence (>2/100 PY). ICCs have been instrumental in reaching MSM and PWID who otherwise do not adequately engage in HIV services due to a variety of reasons (e.g. stigma and discrimination, long travel times to and wait times at public facilities) but these findings indicate community‐based facilities alone are not sufficient to end the epidemic. High incidence tracked with frequently reported risk behaviours—condomless anal sex and sharing of injection paraphernalia. Notably, those younger—especially 21‐ to 25‐year‐olds—were at the highest risk for HIV acquisition within both populations. Among PWID, those more engaged in MOUD—which is delivered daily at the ICC—had significantly lower incidence, highlighting the critical need for easily accessible harm reduction services at community‐based centres to remain a high priority in order to meet global HIV elimination targets.

A global analysis of HIV incidence among MSM found from 1995 to 2010, in many countries, HIV incidence remained high with no evidence of a decline [12]. There were no data from India included but trends in other Asian settings—China and Thailand—suggested increasing incidence. This global analysis reviewed data prior to the timeframe for our analysis. However, similarly, we found no evidence of a decline, rather a consistent increase of 17% each year. Among MSM in Bangkok, Thailand, incidence was nearly two times higher (3.7/100 PY) compared to our findings, though this was from a shorter time period (i.e. 18 months) in 2017−2018 [13]. A recent meta‐analysis estimating HIV incidence for PWID using data from 1987 to 2021 found a global HIV incidence of 1.7/100 PY and, specifically for southeast Asia (inclusive of our Indian data), 3.6/100 PY [14]. Our pooled estimate is similar to the region‐specific estimate though slightly higher at 4.1/100 PY. Though these overall estimates are helpful, they obscure substantial geographic variability and temporal changes. For example, in 2022, incidence in New Delhi in our study was more than six times higher than the average across other cities. Geographic variability in HIV incidence is likely driven by complex structural differences across these cities in terms of culture, history, economics, policies and health service provision, that impact patterns of individual risk behaviours. In terms of temporal trends, when excluding New Delhi from unadjusted analyses, incidence among PWID steadily increased at 6% per year. This is similar to what we have found from cross‐sectional incidence estimates among community‐based respondent‐driven sampling across the same cities; 2.8/100 PY in 2012−2013 and then 5.2/100 PY in 2016−2017 [15].

ICC services—in particular HIV testing—were significantly impacted by the COVID‐19 pandemic in 2020—2021 [11]. We previously examined monthly patterns and found declines in testing for both PWID and MSM associated with COVID waves, more pronounced for the first wave in March 2020. Test positivity (i.e. percentage of conducted tests that were positive) also declined significantly in this period with at least a 1‐year lag before positivity increased. Therefore, estimates for 2020 onwards should be interpreted in light of this change in service utilization. Additionally, there was a large fraction of clients who did not return for repeat testing. Among PWID, those who did not return may have been at greater risk (e.g. more sharing of injecting paraphernalia) conversely, among MSM, those who did not return were likely at lower risk (e.g. less condomless sex). True community‐level HIV incidence is difficult to measure but we previously found facility/clinic‐based incidence methods are likely to underestimate incidence compared to approaches using community‐based samples [16].

## 5 CONCLUSIONS

ICCs have been an integral strategy to identify and engage MSM and PWID but a significant proportion remain under‐engaged in HIV testing and prevention services. This highlights the need to focus on KP in low‐ and middle‐income countries when considering novel approaches to curtail incidence. Such strategies include long‐acting pre‐exposure prophylaxis and optimizing implementation of evidence‐based prevention interventions such as MOUD and syringe service programmes as well as network‐ and peer‐driven approaches to reach those PWID who are less likely to engage in facility‐based programmes.

## COMPETING INTERESTS

The authors declare that they have no competing interests.

## AUTHORS’ CONTRIBUTIONS

AMM, AKS, DDC, SSS, SHM and GML—study concept and design. CKV and SA—data acquisition and analysis; MPG and LG—data analysis and interpretation. All authors have read and approved the final manuscript.

## FUNDING

The study was supported by the National Institutes of Health: R01 DA041034, R01 MH89266, R01 DA032059, K24 DA035684, K23 DA057151, P30 AI060354; Thrasher Research Fund Early Career Award, The Milton Fund (Harvard University).

## Data Availability

The data that support the findings of this study are available from the corresponding author upon reasonable request.
